# UBE4B: A Promising Regulatory Molecule in Neuronal Death and Survival

**DOI:** 10.3390/ijms131216865

**Published:** 2012-12-10

**Authors:** Rami Abou Zeinab, Hong Wu, Consolato Sergi, Roger Leng

**Affiliations:** Department of Laboratory Medicine and Pathology, University of Alberta, Edmonton, AB T6G 2S2, Canada; E-Mails: abouzein@ualberta.ca (R.A.Z.); hwu4@ualberta.ca (H.W.); sergi@ualberta.ca (C.S.)

**Keywords:** UBE4B, p53, p73, p63, nervous system, apoptosis, neurodegenerative diseases

## Abstract

Neuronal survival and death of neurons are considered a fundamental mechanism in the regulation of the nervous system during early development of the system and in adulthood. Defects in this mechanism are highly problematic and are associated with many neurodegenerative diseases. Because neuronal programmed death is apoptotic in nature, indicating that apoptosis is a key regulatory process, the p53 family members (p53, p73, p63) act as checkpoints in neurons due to their role in apoptosis. The complexity of this system is due to the existence of different naturally occurring isoforms that have different functions from the wild types (WT), varying from apoptotic to anti-apoptotic effects. In this review, we focus on the role of UBE4B (known as Ube4b or Ufd2a in mouse), an E3/E4 ligase that triggers substrate polyubiquitination, as a master regulatory ligase associated with the p53 family WT proteins and isoforms in regulating neuronal survival. UBE4B is also associated with other pathways independent of the p53 family, such as polyglutamine aggregation and Wallerian degeneration, both of which are critical in neurodegenerative diseases. Many of the hypotheses presented here are gateways to understanding the programmed death/survival of neurons regulated by UBE4B in normal physiology, and a means of introducing potential therapeutic approaches with implications in treating several neurodegenerative diseases.

## 1. Introduction

With regard to the nervous system, there is no doubt that the regulatory mechanism underlying the growth and death of neuronal cells during early development and at later adult stages is complex [1]. Many aspects of this mechanism have been revealed in normal physiology, but not in pathological situations. In neuronal development, neuronal death is considered to be part of the development mechanism during early stages, in which many neurons are programmed for death, thus avoiding any inappropriate neuronal connections [2]. It has been highlighted that this neuronal elimination mechanism is essential to removing inappropriate differentiated cells after neural precursors undergo exponential proliferation. However, this neuronal elimination is also necessary after differentiated neurons migrate to their anatomical location, in which they establish their target connections [3,4]. At this stage, neurons that do not receive optimal trophic support will undergo apoptosis [5]. Interestingly, neuronal death at later stages has also been characterized as apoptotic in nature in traumatic nervous system injury [6,7]. It is worth mentioning that after surviving the developmental stage, neurons become more stable and less vulnerable to injury, indicating that tight regulation of the apoptotic pathway protects mature neurons and guarantees their survival in the absence of injury [2]. This process opens a gateway for the role of regulatory molecules, which appear to be absent or non-functional at early developmental stages, yet become key factors for monitoring neural death at later stages. The apoptotic nature of neuronal death remained vague until some reports revealed that the mitochondrial death pathway is involved [8]. Such findings drew major attention to the many molecular mechanisms that are involved in the mitochondrial death pathway such as the p53 system and its role in apoptosis [9]. The p53 system is composed of three major proteins: p53, p63, and p73. p53 was the first to be identified, and its role as a tumor suppressor is critical in the field of cancer [10]. Similarly, p73 and p63, which were discovered later, also have tumor suppressor activities [11]. All tumor suppressors have been described as keepers of the genome because they monitor cell proliferation and induce apoptosis, cell cycle arrest, or DNA damage repair [10]. The role of the p53 system in inducing programmed cell death, or monitoring the apoptotic pathway and the significance of the apoptotic death of neuronal cell have suggested the possible links between the p53 system and the nervous system. In the upcoming sections, we will elucidate the role of each protein of these proteins in the nervous system, and we will present the major findings that highlight the consequence of any defects in the regulation of these proteins on the nervous system [12]. First, we will introduce a regulatory molecule that has taken the topic of a wide range of discussions in the last few years, as it is a major regulatory molecule in the p53 system.

In 2011, Wu *et al.* showed that UBE4B acts as an E3/E4 ligase that physically interacts with p53 and MDM2 to promote the polyubiquitination and degradation of p53 in brain tumors, thus decreasing the apoptotic activity of p53 tumor suppressor [13]. Other reports have demonstrated the role of UBE4B in regulating p63 and p73 proteins. Approximately 50%–60% of human tumors show mutations in p53; the remaining tumors exhibit a dysfunctional system despite bearing wild type (WT) [10,11]. The exact mechanism underlying the tight regulation of p53 protein is not fully understood, but it is clear that many processes are involved, including sumoylation [14,15], neddylation [16,17], acetylation and other post-translational modifications [18,19]. However, ubiquitination has been identified as the major regulatory mechanism of p53 protein [20,21]. There are three types of ubiquitination: mono-ubiquitination, multi-ubiquitination, and poly-ubiquitination [22]. In poly-ubiquitination, similar to UBE4B-p53, the proteosomal degradation of the substrate will be induced *post* ubiquitination. UBE4B is a mammalian homolog of the protein UFD2 found in *S. cerversiae*. UBE4B has a conserved U box which confers its ligase activity [13]. As mentioned above, the type of ubiquitination controls the substrate’s fate. For example mono-ubiquitination similar to that induced by the MDM2 E3 ligase does not induce proteosomal degradation *post* ubiquitination [23]. Previous studies have shown that UBE4B is predominantly expressed in mouse neuronal tissues [24], which has attracted increased attention to the UBE4B E3/E4 ligases. In this review, we propose that UBE4B, through its regulatory action on the p53 family proteins, can act as a key factor in the development of the nervous system and can thus be a target molecule in neurodegenerative disease treatments. Also we emphasize on the role of UEB4B in the nervous system via p53-independent pathways, such as axon protection in mice and the clearance of ataxin3, which is responsible for spinocerebellar ataxia type 3.

## 2. The Role of UBE4B in the Nervous System via p53 Family Regulation

### 2.1. UBE4B and p53 in the Nervous System

The association between p53 and tumor inhibition has been confirmed in p53^−/−^ mice, which have a high rate of tumor formation [25]. Although this tumor suppressor activity of p53 is the major focus of a wide range of cancer research, p53 may play a role in the nervous system. According to Armstrong *et al.*, 23% of p53^−/−^ female embryos were characterized with midbrain exencephaly, which results from the abnormal development of the neural tube and the overproduction of neural tissue [26,27]. Therefore, p53 was thought to have two distinct roles: one in tumor suppression and another in nervous system development. Studies analyzing the role of p53 as a transcription factor revealed the apoptotic role of these tumor suppressors by regulating genes involved in the intrinsic mitochondrial death pathway, such as Noxa, Puma, Bax, *etc.*[9]. p53 has also been shown to possess a transcription-independent apoptotic function by acting directly on the mitochondrion [28]. Interestingly, an investigation revealed that the exencephalic phenotype of p53^−/−^ female embryos was similar to the phenotype of animals that had mutations in other members of the intrinsic mitochondrial death pathway, such as caspase 3 and 9, and Apaf-1 [29,30–32]. In addition, it is important to highlight that p53 overexpression induces the death of sympathetic neurons [33]. However, these p53 knockout sympathetic neurons were able to survive when neural growth factors were removed, confirming the role of pro-apoptotic activity of p53 in the early development of the nervous system [34]. The same outcome was obtained when p53 was present but blocked after binding to E1B55K protein [35]. Many other tumor suppressor proteins play a critical role in the apoptotic pathway, and studies have shown that mutations in specific target genes of p53 result in massive levels of apoptosis in the embryonic nervous system [2]. *In vivo* analyses showed that sympathetic neurons during the first two postnatal weeks showed a decrease but not an abolishment of the rate of apoptosis in p53^−/−^ mice [35,36]. Moreover, the role of p53 is not limited to embryonic stages. Less is known about this aspect; however, p53 overexpression has been reported in many neurodegenerative conditions, including seizure-induced excitotoxic damage, middle cerebral artery occlusion, traumatic brain injury, and peripheral nerve injury as it is the case in spinal cord anterior horn cells injury [37,38]. Lastly, p53 neuronal death role was also associated with a wide range of neurodegenerative diseases that are characterized by progressive neuronal death including: Parkinson’s disease, Huntington’s disease, Alzheimer’s disease, *etc.*[1].

All of these findings have drawn attention to the regulatory molecules that inhibit or trigger the expression of p53, particularly in neurodegenerative diseases in which p53 has been reported to be upregulated [39]. In the normal body system, p53 protein is continuously repressed until needed. In DNA damage, p53 repression ceases, allowing p53 to function effectively [40]. This finding indicates that defects in the nervous system may occur with any disruption in the tight regulation of p53, resulting in uncontrolled p53 apoptotic activity. UBE4B (known as Ube4b or Ufd2a in mouse) was recently identified as an E4 ligase that is essential for MDM2 to mediate the polyubiquitination and degradation of p53 [13]. Moreover, Ube4b^−/−^ mice are embryonically lethal, diminishing the possibility that other ligases compensate for the role of Ube4b in p53 regulation [41], which is also supported by rare mutations of other E3 ligases, and excluding their role in p53 regulation at these stages [25]. E3 ligases include MDM2, which was shown to be essential for UBE4B-p53 regulation. Most important, because p53 overexpression is commonly detected in neurodegenerative diseases, defects in UBE4B regulatory mechanism are expected. Accordingly, future extensive investigations should focus on UBE4B expression and activity at the early embryonic stages to reveal how p53 proteins regulate neuronal death and development. Defects in this regulatory mechanism are not limited to the molecular level of UBE4B-p53 interaction; indeed, *post* ubiquitination errors might also exist. For instance, lysine residues involved in UBE4B-p53 ubiquitination are important because lysine chains have a major effect on the *post* ubiquitination fate of the substrate [42]. One example is E3 ligase Pirh2, which ubiquitinates different lysine residues in p53 and p73. Therefore, its degrading effect differs depending on the lysine residue used. Despite degrading p53, Pirh2 does not have the ability to degrade p73, due to utilizing Lys63 [43]. Thus, more investigations should be performed regarding the lysine residues utilized by UBE4B in p53 ubiquitination.

### 2.2. UBE4B and p73 in the Nervous System

As mentioned earlier, many clues on the role of other transcription factors are associated with the role of p53 in sympathetic neuron developmental death. p73 has been suggested to play a role in the nervous system. Many studies have shown the function of p73 as a tumor suppressor; in particular, p73 transactivates a large number of p53 target genes such as *p21* and *Bax*[44]. Interestingly, it has been demonstrated that p53 tumor suppressor activity depends on the presence of p73, whereas the opposite is not required [23,45,46]; p73 is sufficient to induce apoptosis in the absence of p53 [47]. However, it is important to note that unlike p53^−/−^ mice, p73^−/−^ mice do not develop spontaneous tumors [45], but p73^−/−^ mice show significant neuronal abnormalities, such as the loss of peripheral sympathetic neuron, hippocampal dysgenesis, and the majority dies before they are four weeks old [25,45]. In addition, p73^−/−^ SCG (superior cervical ganglion) models show a significant decrease in the number of sympathetic neuron number in late embryogenesis, suggesting an anti-apoptotic role for p73; these findings were confirmed in cultured and *in vivo* analyses [48]. The role of p73 is not limited to embryonic neuronal development, as adult p73^+/−^ sensory neurons were more vulnerable to death compared to wild-type neurons following axonal injury [49]. Furthermore, p73^−/−^ mice that survive after birth develop thin cortical hemispheres and enlarged ventricles [50]. In 2004, Wilson *et al.*, reported an alteration in the subcellular distribution of p73, which accumulated in the nucleus and localized to neurites and neurofibrillary tangles in Alzheimer patients [51]. However, little is known about the role of p73 in neurodegenerative diseases and the existence of p73 isoforms further complicates the situation.

Unlike p53, p73 proteins exist in different naturally occurring isoforms in the human body. TAp73 isoforms appear to mimic the role of p53 in activating similar downstream genes that are involved in cell cycle arrest and apoptosis [50]. However, this is not the case for ΔNp73, which lacks the NH_2_-terminal transactivation domain. Not only do ΔNp73 proteins not play any role in apoptosis, it was proven that they possess a dominant negative “anti-apoptotic” behavior in contrast to the tumor suppressor function of p53 and full length p73 [50,52]. Reports revealed a negative feedback loop between p73 and ΔNp73 in regulating cell death and survival [53,54]. Further analyses at the molecular level showed that the predominant form of p73 in developing brain and sympathetic ganglia is ΔNp73 [48]. This finding led to the conclusion that ΔNp73 is essential for developing both the central and peripheral nervous system because it rescues neurons from p53 apoptotic activity, and it is characterized as a pro-survival protein [55]. Additionally, the overproduction of ΔNp73 in tumor cells blocked chemotherapy induced apoptosis [56]. Many hypotheses have been proposed regarding the mechanism by which ΔNp73 oppose p53 and p73 apoptotic activity and the signals for such pathways. Pozinak *et al.* reported the binding of ΔNp73 to p53 to block its apoptotic activity [48], and Irwin *et al.* proposed that this truncated isoform has the ability to bind the TAp73 isoform, which acts similarly to p53, thus abrogating its apoptotic role [50]. Nevertheless, ΔNp73 regulation has remained unclear. In 2005, a study performed by Hosoda *et al.* revealed that UBE4B binds to p73α but not p73β; UBE4B also induced p73α proteosomal degradation without promoting ubiquitination [57]. However, these results do not negate the interaction of UBE4B with other p73 isoforms, such as ΔNp73. Furthermore, the SAM domain, which has been reported to be essential for UBE4B binding to p73α, is preserved in ΔNp73α [58]. The lack of p73α ubiquitination when interacting with UBE4B raises some questions regarding the degradation process; although it has been reported that the E3/E4 ligases regulate the expression of their substrate target molecules independent of ubiquitination [57]. Accordingly, we cannot disregard the fact that UBE4B can act as an E3/E4 ligase to regulate proteins with apoptotic functions in a ubiquitin-dependent or -independent manner; this hypothesis requires further investigation. Clearly, ΔNp73 was given more attention after it was shown that the neuronal apoptosis observed in p73^−/−^ mice is only partially rescued by the absence of p53 [59], indicating that tight regulation of p73 is not dependent on p53 expression or inhibition. Other major ligases, such as Pirh2, were also reported to bind to and ubiquitinate p73, but cannot induce degradation [43]. For MDM2, the interaction with p73 is at the *N*-terminal transactivation domain, which is absent in the ΔNp73 isoform [23]. Therefore, UBE4B might be a regulatory factor that manipulates the ratio of apoptotic p73 to the anti-apoptotic isoform, but the process at the p73 level is more complicated compared to p53. The role of other cofactors is highly likely, particularly because UBE4B can only degrade alpha isoform but not the beta isoform, which is triggered without any ubiquitination. Even if the fate of the substrate is degradation, the lack of ubiquitination raises some questions regarding the role of UBE4B as an E4 ligase towards p73 and its isoforms.

### 2.3. UBE4B and p63 in the Nervous System

The role of p63 in the nervous system was first proposed by Flores *et al.* who showed that p63 can promote apoptosis in cell lines and act as a pro-apoptotic protein in central nervous system development when DNA damage is induced by gamma radiation [46]. The major finding describing the role of p63 in the nervous system was reported by Kaplan *et al.*, in which the TAp63 isoform was revealed to be the predominant form in the nervous system, particularly in the developing cortex, and the high levels of this isoform were associated with the level of apoptosis in sympathetic neurons [60]. In addition, TAp63γ overexpression caused neuronal apoptosis even when NGF was present. Further findings supported the pro-apoptotic role of p63; p63^−/−^ cultured neurons showed significant resistance to apoptosis following NGF withdrawal [61]. *In vivo* analysis also revealed that embryonic p63^−/−^ mice developed defects in naturally occurring neuron death, and all died at birth [62]. As mentioned above, p63 and p53 targets similar apoptotic genes to induce apoptosis, and both of proteins increase Bax gene expression, which is essential for the neuronal apoptotic activity of these proteins. As discussed above, Bax triggers the mitochondrial apoptotic pathway [61]. However, in the same study, the TAp63 apoptotic activity was found to be independent of p53. This result was later confirmed by Gressner *et al.*, who revealed that TAp63 can mediate apoptosis via other death receptor complexes such as CD95, TNF, and FLIP [63] which is in contrast to the p53 requirement of the presence of p63 to successfully perform its apoptotic activity. Cells deficient in both p63 and p73 exhibit a significant resistance to neuronal apoptosis despite the presence of functional p53. Thus, p53 is proposed to operate upstream of p63 and p73 and cannot trigger cell death by itself as proposed by Nictorea *et al.*[62]. Similar to p73, the ΔNp63 isoform, which contains the NH2-terminal transactivation domain, has also been reported to act as an anti-apoptotic protein by promoting cell growth and proliferation [64]. Although little is known about the role of ΔNp63 compared to ΔNp73, it has been shown that ΔNp63 is overexpressed in squamous cell carcinomas [65–67], and it is believed to enhance cell growth by blocking p53 mediated transactivation [64]. These findings were confirmed when the expression of p53 target genes, such as *p21*, *Noxa*, and *Puma*, was stabilized when ΔNp63 is deleted [68]. ΔNp63α was reported to be inhibited after UV and paclitaxel treatment [69], and thus, ΔNp63 is speculated to act as a key factor blocking TAp63, particularly because it has been revealed to be more stable than TAp63 [70]; further investigation is required to examine this possiblity. The similarities between p63 and p73 in terms of their isoform functions, in which the TA isoform possess apoptotic function and the ΔN isoform counteracts this function, was thought to aid a better understanding of the entire regulatory mechanism.

Unfortunately, with regard to UBE4B, the mechanism is more complex. First, the only isoform of p63 found to be regulated by UBE4B was ΔNp63α, whereas TA isoform showed no link to UBE4B [68]. UBE4B binds to and stabilizes ΔNp63α, and stabilization was first noted by inhibiting ubiquitination; therefore, the degradation of ΔNp63α is a cisplatin-induced mechanism. When ectopically expressed, UBE4B efficiently extends the half-life of ΔNp63α [68]. However, the molecular mechanism underlying this regulatory pathway remains unknown. As a first step, we propose an investigation of the UBE4B-ΔNp73 relationship. Second, the fact that UBE4B has no relation to any of the the β isoform of either p63 or p73 might also explain the specificity of this ligase. In conclusion, the ratio of expression of the p53 family proteins, including those possessing apoptotic activity or anti-apoptotic activity, is the key factor in maintaining a stable developmental mechanism for the nervous system. Therefore, regulatory molecules that affect protein expression and activity, such as UBE4B, will be a turning point in the field of neuronal physiology and neurodegenerative disease.

## 3. The Role of UBE4B in the Nervous System Is Independent of p53 Family Regulation

Another characteristic of neurodegenerative diseases is the presence of insoluble aggregates in the neurons due to polyglutamination [71], which is common in Huntington disease, spinobulbar muscular atrophy, dentatorubral-pallidoluysian atrophy, spinocerebellar ataxia, Alzheimer disease, and Parkinson disease [72,73]. The intracellular aggregates become conjugated with ubiquitin, altering the conformational structure of target proteins [74]. Because these intracellular aggregates are associated with ubiquitin, all E3/E4 ligases regulating the ubiquitination mechanism become target molecules in this process [74]. However, because these aggregates are detected in neurons [75] and Ube4b is the only ligase expressed predominantly in the neural tissue of adult mice [24], many scientists have speculated on the role of Ube4b in this process. These speculations were also supported by other observations, such as the lethality of Ube4b double deletion in mice; Ube4b^+/−^ mice also displayed axonal dystrophy in the nucleus gracilis and the degeneration of Purkinje cells in endoplasmic reticulum stress [76]. In parallel, when Ube4b was knocked down, the level of polyubiquitination was remarkedly decreased. Matsumoto *et al.* were the first to show the role of Ube4b in polyubiquitinating and degrading ataxin3, whose abnormal expansion of the polyglutamine tract causes spinocerebellar ataxia type 3 [74]. Ube4b showed no difference in the level of ubiquitination in normal or pathological ataxin3, which has an expanded polyglutamine tract; however, this process was shown to be mediated by VCP proteins [74,77]. It was proposed that VCP, ATPase valosin-containing protein [78], mediates the dissociation of Ube4b from ataxin3, inducing its degradation. This dissociation mechanism is blocked for pathological ataxin3, despite polyubiquitination of ataxin3 by Ube4b. Interestingly, VCP, which has previously been shown to exhibit no ligase activity, was proven to be associated with Ube4b and not with any other E3 ligases [74]. Based on that finding, Ube4b is considered to be a rate-limiting factor in mediating the ubiquitination and polygutamine aggregation in neurodegenerative diseases.

Axon degeneration is a consistently common phenotype for many neurodegenerative disorders [79]. Nerve injury, such as lesions, vincristine neuropathy, and myelin-related axonopathies, is always accompanied by direct axon degeneration within two days of the stimulus [80–82]. The Wallerian degeneration process, a non-apoptotic death program, has been shown to chiefly regulate axon degeneration in response to injury [83]. Wallerian degeneration has been proposed to play a prominent causative role in a wide range of human neuropathologies in trauma, spinal cord injury or even at early stages [84,85]. Although little is known about Wallerian degeneration and the signals that initiates this pathway, a spontaneous dominant mutation delays Wallerian degeneration ten-fold and is known as slow Wallerian mutation (Wld^s^) [80,84]. As a result, scientists have concluded that Wallerian degeneration is not a passive process but an active regulated process [86–88]. Furthermore, animal models highly support this hypothesis, and a delay in axonal and even synaptic degeneration was reported in Wld^s^ mice [89,90]. As in mice, Wld^s^ rats display axon survival up to two weeks after transection and remain functional for at least one week [91]. The progression of many diseases, such as axonal injury, Parkinson’s disease, and cerebral ischemia, was also altered in Wld^s^[80,84,92]. Further analysis revealed a significant role of Wld^s^, which dominantly delays Wallerian degeneration 10-fold [93], findings have been confirmed *in vitro* and *in vivo*[83]. Interestingly, the Wld^s^ gene encodes chimeric protein composed of 70 amino acids of Ube4b linked to full length nicotinamide mononucleotide adenylyl transferase 1 (Nmnat1) [94]; both moieties have been shown to be essential for the proper function of the Wld^s^ protein in delaying degradation [79,94]. Importantly, this chimeric protein is missing in wild-type mice [95]. Although the Ube4b region essential to Wld^s^ does not contain the U box, it has been demonstrated that Ube4b moiety influences the intracellular distribution of the covalently attached Nmnat1 and, consequently, the distribution of nuclear NAD^+^ synthesis machinery [96]. The same portion of Ube4b has been demonstrated to bind to the VCP protein; 16 of the 70 amino acids form the VCP binding motif. This binding between Ube4b N portion and VCP influences the redistribution of molecules in nuclei *in vivo* and *in vitro* when Wld^s^ is present [97,98]. Many functions of VCP have been identified, little is known about the role of VCP with regards to the Wld^s^ protein and the delay in neural degeneration. However, the role of VCP in Ube4b-ataxin3 dissociation and the binding of Wld^s^ proteins through the Ube4b N portion focus more attention on the role of VCP in neuronal regulation. This role is exclusively associated with Ube4b because VCP has no association with any other ligases. Further investigation is necessary to elucidate the exact role of VCP. The lack of a chimeric protein raises some possibilities that the Ube4b portion might be mutated or altered, thus abolishing the formation of the chimeric protein, and further investigations are needed to clarify this as well.

## 4. Conclusions

**Figure 1:**
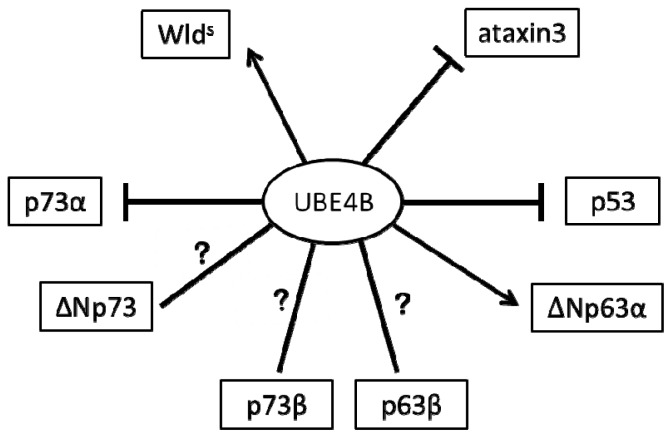
Schematic model for UBE4B showing the inhibition (⊣), stabilization (→), or possible relation (?) with p53 family proteins, Wallerian pathway, and polyglutamination in neurodegenerative diseases.

In conclusion, UBE4B is involved in multiple pathways that are all associated with neuronal survival and degradation. Whether through the p53 family or other processes, UBE4B is definitively implicated in neuronal survival. A model for the role of UBE4B is summarized in the Figure 1. Investigating the exact role of UBE4B and all cofactors associated with its function could contribute to understanding the normal physiology of the nervous system and also be a gateway to many therapeutic and pharmaceutical approaches that aim to treat neurodegenerative diseases by protecting neurons from death as a response to mutation or injury.
